# Patients from the Oral Oncology Center, UNESP, Araçatuba with an indication for prosthesis

**DOI:** 10.3892/mco.2013.105

**Published:** 2013-04-17

**Authors:** ALDIÉRIS ALVES PESQUEIRA, MARCELO COELHO GOIATO, DANIELA MICHELINE DOS SANTOS, AMÁLIA MORENO, MARCELA FILIÉ HADDAD, PAULA DO PRADO RIBEIRO, LISIANE CRISTINA BANNWART, GLAUCO ISSAMU MIYAHARA

**Affiliations:** 1 University Sagrado Coração (USC), Bauru, SP 17011-160;; 2 Department of Dental Materials and Prosthodontics, São Paulo State University (UNESP), Araçatuba, SP 16015-050, Brazil;; 3 Oral Oncology Center, School of Dentistry, São Paulo State University (UNESP), Araçatuba, SP 16015-050, Brazil

**Keywords:** head and neck neoplasms, quality of life, age groups, neoplasms by site, gender identity

## Abstract

Head and neck tumors are a major health concern worldwide, due to their high incidence and mortality rates, particularly in developing countries. In Brazil, this type of cancer is commonly diagnosed and studies suggested that it may be the leading cause of mortality in the country. The increase in life expectancy worldwide, as well as environmental and behavioral factors, are related to carcinogenesis. Therefore, an understanding of basic epidemiology and statistical methods is critical, in order to promote early diagnosis and cancer prevention. Cancer patients with an indication for prosthesis were selected from the medical records of the Oral Oncology Center, School of Dentistry, São Paulo State University (UNESP), Araçatuba, between 1991 and 2010. The following variables were recorded: gender, age, type and location of the lesion, radiation dose and dental prosthesis. The majority of the patients were male (74.15%) and >60 years of age (53.37%). Tumors were most commonly located in the floor of the mouth (11.1%) and squamous cell carcinoma was the most prevalent type (72.8%). This study provides the profiles of patients who attended the Oral Oncology Center and the results may aid in the creation of cancer prevention programs.

## Introduction

Approximately 10% of head and neck tumors are malignant and affect multiple sites. Of these, 40% are located in the oral cavity, 25% in the larynx, 15% in the pharynx, 7% in the salivary glands and 13% in other locations. Over 8 million new cases are diagnosed annually, of which 212,000 originate in the oral cavity (1).

Currently, cancer is the second cause of disease-related mortality in Brazil and studies suggested that cancer-related mortality rates may exceed cardiovascular disease-related mortality rates (2,3). Compared to cancer located in other organs, head and neck cancer is relatively common and this anatomical location has the fourth and seventh highest incidence rates among males and females, respectively. In Brazil, >85% of the cases are diagnosed at an advanced stage, which reduces survival expectancy and requires a more aggressive therapeutic approach, leading to severe deformities and significantly compromising the quality of life of the patients after treatment (4).

Cancer is a multifactorial disease and is attributed to external or internal factors, which may be interlinked. External factors are associated with the environment, as well as the cultural and social habits of the patient. Internal factors may be systemic, general and genetic. The ability of the body to defend itself from external aggression is another potential factor. These factors may interact in several ways, increasing the likelihood of malignant transformation of normal cells (2,5).

Surgery with or without radiotherapy and chemotherapy is the most common therapeutic approach in the treatment of cancer. The basic principle of cancer surgery involves tumor resection with wide safety margins, which frequently leads to large maxillofacial defects and affects the patient functionally, aesthetically and psychologically. Early diagnosis, combined with currently available treatments (radiotherapy, chemo-therapy, surgery and bone marrow transplantation), increases survival rates and improves the quality of life of patients with advanced, incurable cancer (1,2).

Therefore, in addition to tumor resection, it is imperative to rehabilitate patients and preserve their quality of life. Maxillofacial prostheses promote a rapid psychosocial reintegration of patients, are cost-effective, reduce hospitalization time and facilitate the inspection of the affected area (6,7).

The aim of this study was to assess the epidemiological data of cancer patients with an indication for prosthesis who attended the Oral Oncology Center, School of Dentistry, UNESP, Araçatuba, between 1991 and 2010.

## Materials and methods

The present study was approved by the Human Research Ethics Committee of UNESP, Process FOA/2008-01688.

Patients diagnosed with cancer and an indication for prosthesis were selected from the medical records of the Oral Oncology Center, UNESP, Araçatuba, between 1991 and 2010. Data were reviewed and the variables used in the study were included. A total of 178 medical records were used. Patient consent was obtained from the patient and the patient’s family.

Detailed information regarding irradiated patients and radiation dose were not assessed, since a large number of patients had undergone radiotherapy treatment in centers located outside of Araçatuba and that information was not provided by the medical records. Therefore, we registered the responses to this question as ‘yes’, ‘no’ or ‘treated elsewhere’. Following data collection, the results were analyzed.

## Results and Discussion

Data obtained from the present epidemiological study are presented in Tables I–V.

### Gender and age

Table I shows the percentage values regarding patient gender. The majority (74.15%) of cancer patients were male. Of note, men were more exposed to environmental factors and often presented with backgrounds of smoking and alcohol consumption, which may explain our results. The findings of the present study corroborate those of previous studies which demonstrated a higher incidence rate of head and neck cancer among males (2,8,9). Data obtained from a previous study conducted by Jemal *et al* (10) demonstrated the same trend in the United States, where 1 in 2 men has a higher risk of cancer development, compared to 1 in 3 women.

A previous study conducted by the National Cancer Institute (NCI) (5) demonstrated that tumors were responsible for 12.73% of the 946,392 deaths reported in 2000 in Brazil, with 53.97 and 46.01% of cancer-related deaths occurring in men and women, respectively.

A previous study by Goiato and Fernandes (7) reported that of the 88 cases of laryngeal cancer diagnosed at the Oral Oncology Center in Araçatuba, 80 were men.

According to the NCI (2), oral cancer is the eighth most common cancer among Brazilian men (9,985 estimated cases per year) and the ninth among Brazilian women (3,895 estimated cases per year).

In this study, the age variable was divided into 3 groups (Table I): 0–45, 46–60 and >60 years. Subjects >60 years of age were the most commonly affected by cancer (53.37% of cases). These data are in agreement with the findings of Carvalho *et al* (8), according to which 75% of oral cancers were diagnosed in patients >60 years of age.

The increasing number of individuals >60 years of age has led to an increase of chronic diseases associated with longer exposure to risk factors, such as cardiovascular disease, obesity, diabetes, hypertension and cancer, since only 5–10% of all cancers are hereditary (individual susceptibility). Approximately 80–90% of cancers are associated with environmental factors, such as smoking and occupational activities (lung cancer), excessive sun exposure (skin cancer), alcohol consumption (gastric cancer) and excessive consumption of fatty foods (cancer of the colon and rectum) (2,5). According to Jemal *et al* (10), 77% of all cancers are diagnosed in individuals >55 years of age.

### Irradiated patients and radiation dose

Table II shows the distribution of irradiated patients and the radiation doses. The majority of patients (65.1%) received radiotherapy treatment, whereas only 2.2% did not; the remaining 32.7% had undergone treatment in centers located elsewhere.

Radiotherapy is considered a well-established method of treating or managing malignant head and neck tumors and aims to eradicate tumor cells and preserve the surrounding normal tissue included in the irradiation field (11). Radiotherapy is currently considered the most effective curative cancer treatment next to surgery (1). According to Burnet *et al* (13), ∼60% of patients affected by tumors are likely to be subjected to radiotherapy with curative intent, either alone or in combination with surgery or chemotherapy. Delay in initiation of radiotherapy and treatment interruptions, resulting in prolongation of the total treatment time, are critical factors affecting disease control (2,13).

The oral cavity is prone to radiotherapy complications (12). According to Parliament *et al* (14), the mandible may receive high doses of radiation, although the maximum dose should not exceed 7,500 Gy.

In this study, 44.8% of irradiated patients received radiation doses >5,000 cGy. Conventional radiotherapy treatments are commonly divided into 5 doses of 180–200 cGy/day, with a total dose of 5,040–7,000 cGy, administered during a time period of 2–8 weeks without interruption, alone or as a complementary treatment, depending on the tumor stage; however, variations may occur (15).

Dentists should familiarize themselves with the currently available protocols for patient management, in order to avoid further complications such as osteoradionecrosis, dryness of the mouth, altered taste and mucositis, which may directly affect the quality of prosthetic rehabilitation of these patients.

### Type of cancer

Table III shows the percentage values of different types of cancer. Squamous cell carcinoma had the highest incidence (72.8% of cases), followed by basal cell carcinoma (4.0%), melanoma (2.8%), cystadenocarcinoma (2.2%) and adenocarcinoma (1.7%).

Among oral cancers, squamous cell carcinoma is the most prevalent type. Its geographical distribution varies in different regions of the world; therefore, it is imperative to determine its pathogenetic as well as its epidemiological profile. Our findings are in accordance with those of Rosa *et al* (16), who investigated 1,228 cases of oral cancer in the Napoleão Laureano Hospital (João Pessoa, PB, Brazil) over 15 years and reported that squamous cell carcinoma was the predominant lesion (88.8%). Furthermore, the literature reveals that >90% of head and neck cancers are of the squamous cell type (9,17).

### Tumor location

Table IV shows the most prevalent tumor locations among patients of the Oral Oncology Center, UNESP. The floor of the mouth had the highest incidence rate (11.1%), followed by the tongue (10.6%), palate and oropharynx (7.3%).

In a previous study conducted by Sannomiya *et al* (19) regarding patient distribution according to tumor location in various regions of the oral cavity, the tongue was the anatomical area with the highest incidence rate, with a total of 563 cases (54% of the total sample), followed by the floor of the mouth with 111 cases (11%).

According to Sampaio *et al* (20), of the 236 cases of oral squamous cell carcinoma biopsies registered in the Department of Pathology, UNESP, 26.3% were located in the floor of the mouth, 25.8% in the gingiva or alveolar ridge and 17.8% in the tongue.

Rosa *et al* (16) also demonstrated a higher prevalence of oral cancer in the tongue and floor of the mouth.

### Prostheses

Table V shows the different types of prostheses used by oral cancer patients, among which complete dentures had the highest prevalence (81.8%). This result may be due to the fact that the majority of the patients were >60 years of age (53.37%).

In a retrospective study on the risk of cancer of the upper airway, the use of dentures was not associated with an increased incidence of cancer. However, a history of secondary oral ulcers due to poor denture adjustment and poor oral hygiene (low frequency of toothbrushing) was related to higher cancer risk. Poor oral hygiene may allow for longer contact beween carcinogens (e.g., tobacco or alcohol) and the mucosa (3). However, to the best of our knowledge, no study has confirmed this hypothesis.

Dentures must be inspected and well-adjusted in order to avoid mucosal irritation. Patients must be instructed to remove the dentures in case of mucosal ulceration (21).

Based on the results of this study, we concluded that the patients treated at the Oral Oncology Center, School of Medicine, UNESP, Araçatuba, were predominantly men, >60 years of age. Several improvements in the information system are required in order to obtain more reliable data and to improve the public health system. This study has provided the profile of patients who attended the Oral Oncology Center and may aid in the creation of cancer prevention programs.

## Figures and Tables

**Table I.** Patient distribution according to gender and age (absolute no. and percentage of cases).

Variable	No. of patients (%)
Gender	
Male	132 (74.15)
Female	46 (25.85)
Age (years)	
0–45	16 (8.98)
46–60	67 (37.65)
>60	95 (53.37)

**Table II.** Patient distribution according to irradiation and radiation dose (absolute no. and percentage of cases).

Variable	No. of patients (%)
Irradiated patients	
Yes	116 (65.1)
No	4 (2.2)
Treated elsewhere	58 (32.7)
Radiation dose	
>5000 cGy	52 (44.8)
<5000 cGy	1 (0.9)
Treated elsewhere	63 (54.3)

**Table III.** Percentage of different types of cancer.

Type of tumor	Percentage (%)
Squamous cell carcinoma	72.8
Basal cell carcinoma	4.0
Melanoma	2.8
Cystadenocarcinoma	2.2
Adenocarcinoma	1.7
Other	16.5
Total	100

**Table IV.** Percentages of different tumor locations.

Location	Percentage (%)
Floor of mouth	11.1
Tongue	10.6
Palate	7.3
Oropharynx	7.3
Lips	6.2
Other	57.5
Total	100

**Table V.** Absolute values and percentages of the different types of prosthesis used by oral cancer patients.

Type of prosthesis	No of patients (%)
Complete dentures	148 (81.78)
PRD	18 (9.95)
Fixed partial dentures	2 (1.10)
Overdentures	1 (0.55)
Complete dentures + PRD	4 (2.21)
Obturators dentures	2 (1.10)
Nasal prosthesis	1 (0.55)
Tongue prosthesis	1 (0.55)
Oculopalpebral prosthesis	4 (2.21)
Total	181 (100.00)

PRD, partial removable dentures.
